# Microbiome modulation of implant-related infection by a novel miniaturized pulsed electromagnetic field device

**DOI:** 10.1038/s41522-025-00667-0

**Published:** 2025-02-26

**Authors:** João Gabriel S. Souza, Fabio Azevedo, Maria Helena Rossy Borges, Raphael Cavalcante Costa, Takahiko Shiba, Shlomo Barak, Yaniv Mayer, Luciene Cristina de Figueiredo, Magda Feres, Valentim A. R. Barão, Jamil A. Shibli

**Affiliations:** 1 Department of Periodontology, Dental Research Division, Guarulhos University, Guarulhos, São Paulo, 07023-070 Brazil; 2 Department of Prosthodontics and Periodontology, Piracicaba Dental School, Universidade Estadual de Campinas (UNICAMP), Piracicaba, São Paulo, 13414-903 Brazil; 3 School of Dentistry, Alfenas Federal University, Alfenas, Brazil; 4 Department of Oral Medicine, Infection, and Immunity, Harvard School of Dental Medicine, Boston, Massachusetts, 02115 USA; 5 Department of Periodontology, Rambam Health Care Campus, Haifa, Israel

**Keywords:** Applied microbiology, Dentistry

## Abstract

Dental implant-related infections, which lack effective therapeutic strategies, are considered the primary cause for treatment failure. Pulsed electromagnetic field (PEMF) technology has been introduced as a safe and effective modality for enhancing biological responses. However, the PEMF effect on modulating microbial diversity has not been explored. Thus, we tested a miniaturized PEMF biomedical device as a healing component for dental implants. PEMF activation did not alter the chemical composition, surface roughness, wettability, and electrochemical performance. PEMF effectively controlled chronic in vitro polymicrobial microbial accumulation. The in vivo study where devices were inserted in the patients’ oral cavities and 16S RNA sequencing analysis evidenced a fivefold or more reduction in 23 bacterial species for PEMF group and the absence of some species for this group, including pathogens associated with implant-related infections. PEMF altered bacterial interactions and promoted specific bacterial pathways. PEMF has emerged as an effective strategy for controlling implant-related infections.

## Introduction

Implantable devices have gained widespread acceptance in the fields of orthopedics and dentistry, particularly for the prosthetic rehabilitation of absent anatomical structures^1,2^. In the context of oral rehabilitation, dental implant placements stands out as the main therapeutic approach, boasting high success rates and clinical longevity^3,4^. These remarkable outcomes have been attributed to the great chemical, physical, and mechanical properties of the biomaterials used for implant manufacturing, as well as the implementation of enhanced surgical protocols^5^.

However, upon exposure to the oral environment, implant devices become susceptible to microbial adhesion and accumulation, often resulting in an exacerbated inflammatory response and tissue damage^6–8^. In terms of the use of implantable devices, the oral cavity poses a significant challenge due to its hosting of close to a thousand microbial species and frequent exposed to different factors that disrupt the host homeostasis^7^^,9^^,10^. Evidence has highlighted polymicrobial biofilms as the primary factor triggering dental implant-related infections, wherein the inflammation affects the surrounding mucosa, leading to progressive loss of supporting bone^11^. Such implant-related infections have been considered the main reason for dental implant treatment failure, affecting 20–45% of patients^12,13^, imposing a substantial financial burden on both patients and healthcare systems^14^. Unfortunately, there is no consensus regarding the most effective therapeutic approach to treat this condition^15^.

To address these challenges and enhance disease management, biomedical engineering has developed alternative strategies for controlling and treating these conditions, such as surface coatings^16,17^. However, such coatings have shown limited efficacy and have often resulted in some degree of cytotoxicity. In the pursuit of enhancing interaction with biological systems and cellular signaling, bioelectromagnetics has emerged as an interdisciplinary science focused on magnetic, electric, and electromagnetic phenomena associated with living systems^18^. Hence, the electromagnetic field (EF) has arisen as a safe and effective modality for eliminating microbial species adhered to the implant surfaces. EF constitutes a stimulation system generated by the interaction of flowing or alternating electric and magnetic fields moving in tandem through the environment^19^. The magnetic field is generated by the flow of electric charges, which in turn creates the electric field, modulated by the frequency and wavelength applied^20,21^. Distinct parameters of the EF, including type, frequency, and exposure time, have the capacity to modulate biological responses, either by inhibiting or stimulating them^19^. Among the different field types, the pulsating type is characterized by an oscillating stimulation, involving intermittent interruptions in the electric circuits, leading to gaps between the pulses^19^. Pulsed electromagnetic fields (PEMF) have been widely explored in the biomedical field, including for the treatment of cancer^22^, osteoarthritis^23^, and wound healing processes^24^. Laboratorial and clinical evidence have indicated the effects of PEMF on host-cell differentiation and proliferation processes^25–27^. These effects have been attributed to the PEMF effect on membrane-related and intracellular molecules, as well as on specific signaling pathways^27^. Although most evidence has focused on incorporating this technology into metallic biomedical devices due to their inductibility properties, PEMF has also been explored for non-metallic devices with structures that allow field induction, primarily to induce specific cellular responses^22,28^. Furthermore, this technology has also shown significant capacity for antimicrobial activity.

PEMF has also been explored as a potent approach for controlling and treating biofilm-related infections^29^. Although its antimicrobial mechanisms are poorly understood, PEMF has demonstrated effective antibacterial abilities against some microbial species, including *Streptococcus epidermidis*^29^, *Escherichia coli*^30^, and even multidrug-resistant pseudomonas biofilm^31^. Moreover, PEMF has been tested in vitro as an adjunctive therapy administered with antibiotics, showing potential for improving bacterial killing^29,32^. Previous evidence has indicated that PEMF, when used as a healing device for dental implants, reduced the levels of 7 bacterial species in an in vitro multi-species biofilm model^33^. However, the antimicrobial effect of PEMF on a biofilm model that considers the progressive microbial accumulation and diverse microbiome composition of the entire oral environment remains unexplored. Furthermore, the effect of the PEMF activation on the surface properties and electrochemical behavior, which are important parameters for biomedical devices, has not been explored. Therefore, we sought to evaluate the impact of a miniaturized PEMF biomedical device on controlling and modulating polymicrobial biofilm accumulation and diversity, in addition to evaluating the physical, chemical, and electrochemical properties of the material. Through a comprehensive in vitro and in vivo evaluation, this study seeks to provide valuable insights into the potential clinical application of PEMF technology for enhancing dental implant success rates and mitigating implant-related infections. Furthermore, for the first time high-throughput techniques was applied to unravel the PEMF effect on microbiome composition. Our study also sheds light on a critical mechanism by which this technology modulates microbial interactions and influences bacterial pathways under conditions that challenge microbial survival.

## Results

**Fig. 1:**
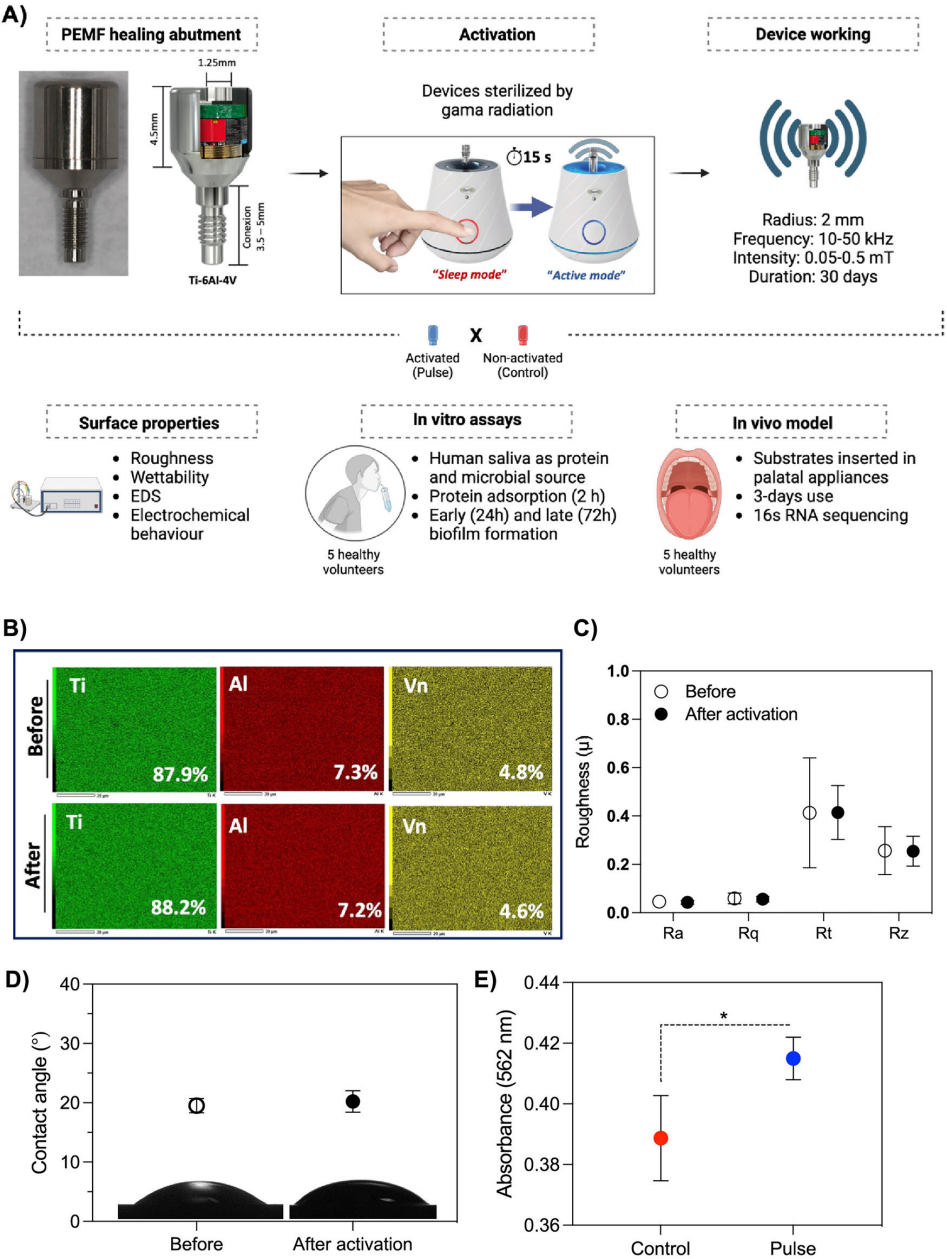
Pulsed electromagnetic field (PEMF) evaluation. **A** Schematic sequence of experimental design. Miniaturized PEMF devices as dental implant healing abutment/component was used. Devices were activated to start pulse emission. Non-activated or device after activation phase (30 days) were used as control. Devices were evaluated in terms of surface properties, in vitro biofilm model and protein adsorption, and in vivo models, where the devices were inserted in the oral cavity of volunteers. **B** chemical composition of the devices before and after the 30 day activation phase evaluated by energy dispersive spectroscopy. **C** Surface roughness of the devices before and after 30 day activation phase. **D** Surface wettability by water contact angle. **E** Salivary protein adsorption on non-activated (control) and activated (pulse) devices. Pool of stimulated human saliva (5 healthy volunteers) was used for protein adsorption (2 h). Total protein quantification was performed using bicinchoninic acid method and absorbances measured. * Indicates statistical difference (*p* < 0.05) by Bonferroni *t*-test.

Biological and microbiological outcomes were compared between activated and non-activated PEMF devices (Fig. 1A). The effect of the PEMF device on modulating biofilm growth and composition was evaluated using in vitro and in vivo models, simulating conditions within the oral environment. Furthermore, effects of pulse activation period (30 consecutive days) on device surface’s properties was evaluated.

### PEMF activation has no impact on the device surface properties

Miniaturized PEMF devices were evaluated for changes in surface properties following the activation phase. Firstly, we compared the changes in the properties of the device after the 30 day activation phase, corresponding to the battery’s duration. The continuous pulse emitted over this period did not impact the chemical composition of the structure (Fig. 1B), nor did it alter its surface roughens and (Fig. 1C) or wettability (Fig. 1D). Overall, the device exhibited a hydrophilic surface (≈20°) and low roughness values (Rt ≈0.4 μm). Interestingly, the pulse group (PEMF activated) demonstrated a significant increase (*p* > 0.05) in salivary protein adsorption on the device compared to the non-activated (control) group (Fig. 1E). Consequently, as anticipated, PEMF activation did not affect the chemical-physical surface properties, but it notably stimulate the crucial biological response, namely, protein adsorption.

### Electrochemical behavior of PEMF device

**Fig. 2:**
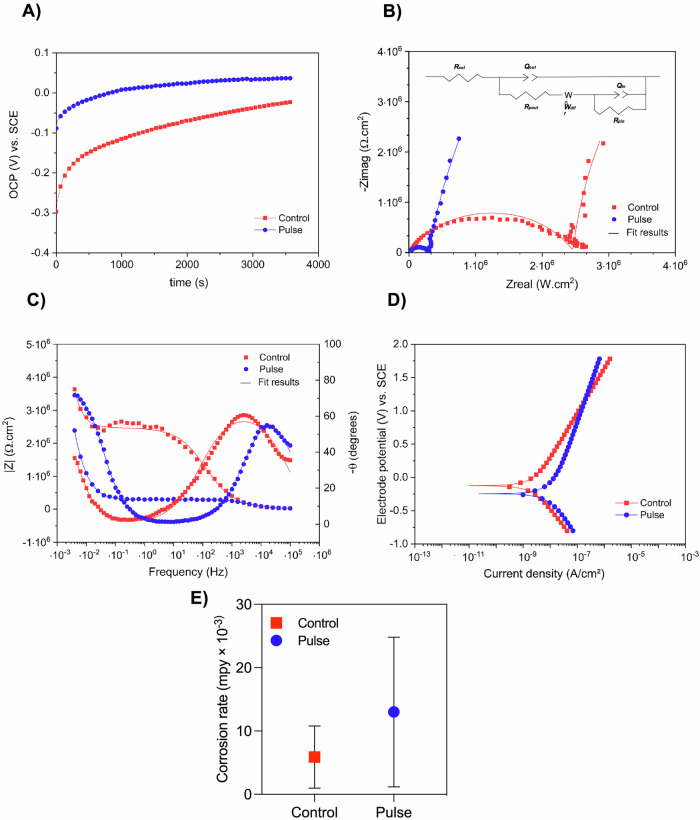
Electrochemical behavior of activated pulsed electromagnetic device (pulse), compared with non-activated (control). Electrochemical tests were conducted for analyzing the corrosion stability of pulsed and control healing abutments in artificial saliva. **A** Open circuit potential analysis, performed for 3600 s to evaluate the free corrosion potential of the material. **B** Nyquist diagram. **C** Bode plots showing variations in impedance as a function of frequency. **D** Potentiodynamic polarization curves. **E** Corrosion rate.

The electrochemical results evidenced that PEMF had no impact on the corrosion resistance and electrochemical performance of the healing devices, confirming its reliability as a treatment modality. In the OCP analysis (Fig. 2A), the pulse group demonstrated nobler potential values (more positive) compared to the control group, indicating a reduced tendency to the corrosion process. EIS was performed to investigate the corrosion kinetics of both pulse and control groups. Bode plots were used to illustrate variations in impedance as a function of frequency (Fig. 2B). Although the control group exhibited slightly higher impedance values at low frequencies, the overall behavioral trend between the two groups was similar. Additionally, in the phase angle plot, both groups demonstrated similar angles of approximately 60 degrees, indicating suitable protective behavior against corrosion (Fig. 2B). In the Nyquist diagram, despite slight differences in the semicircle amplitude, both the pulse and control groups exhibited similar patterns (Fig. 2C). A small capacitive semicircle observed within the high-frequency range may be associated with the primary charge transfer process at the electrode surface. For both groups, data were modeled using equivalent electrical circuits comprising two pairs of elements: R_pout_/Q_out_ and R_pin_/Q_in_, representing the polarization resistance and constant phase element of the outer and inner layer of the oxide film in the healing abutment. In addition, the Warburg diffusion element (W_diff_) was used to depict the phenomenon of substrate diffusion to the electrolyte (Fig. 2C). The chi-square (*X*^2^) values were on the order of 10^-3^, indicating significant agreement between the experimental and simulated EIS data. Overall, both healing devices exhibited a protective behavior towards the surface and an appropriate resistance to ion exchange in artificial saliva. This was supported by the observation that PEMF did not significantly influence the electrical parameters of the pulse group, as evidenced by comparable R_ptot_ and Q_total_ values (p < 0.05) (Supplementary Table 1).

Furthermore, to assess the corrosion resistance performance between the control and pulse groups at different potentials, we conducted potentiodynamic polarization experiments. The potentiodynamic polarization curves (Fig. 2D) revealed similar electrochemical behavior for both groups, indicating the electrochemical stability. Relative to the electrochemical parameters, no difference was found in the corrosion rate between the groups (Fig. 2E), and this trend was also observed for E_corr_, *i*_corr_, and *i*_pass_ data (Supplementary Table 2). Therefore, the findings suggest an equivalent electrochemical performance between the groups, demonstrating that PEMF is a safe strategy for application in biomedical implants.

### PEMF healing device reduced in vitro polymicrobial accumulation and modulated biofilm composition towards a profile compatible with health

**Fig. 3:**
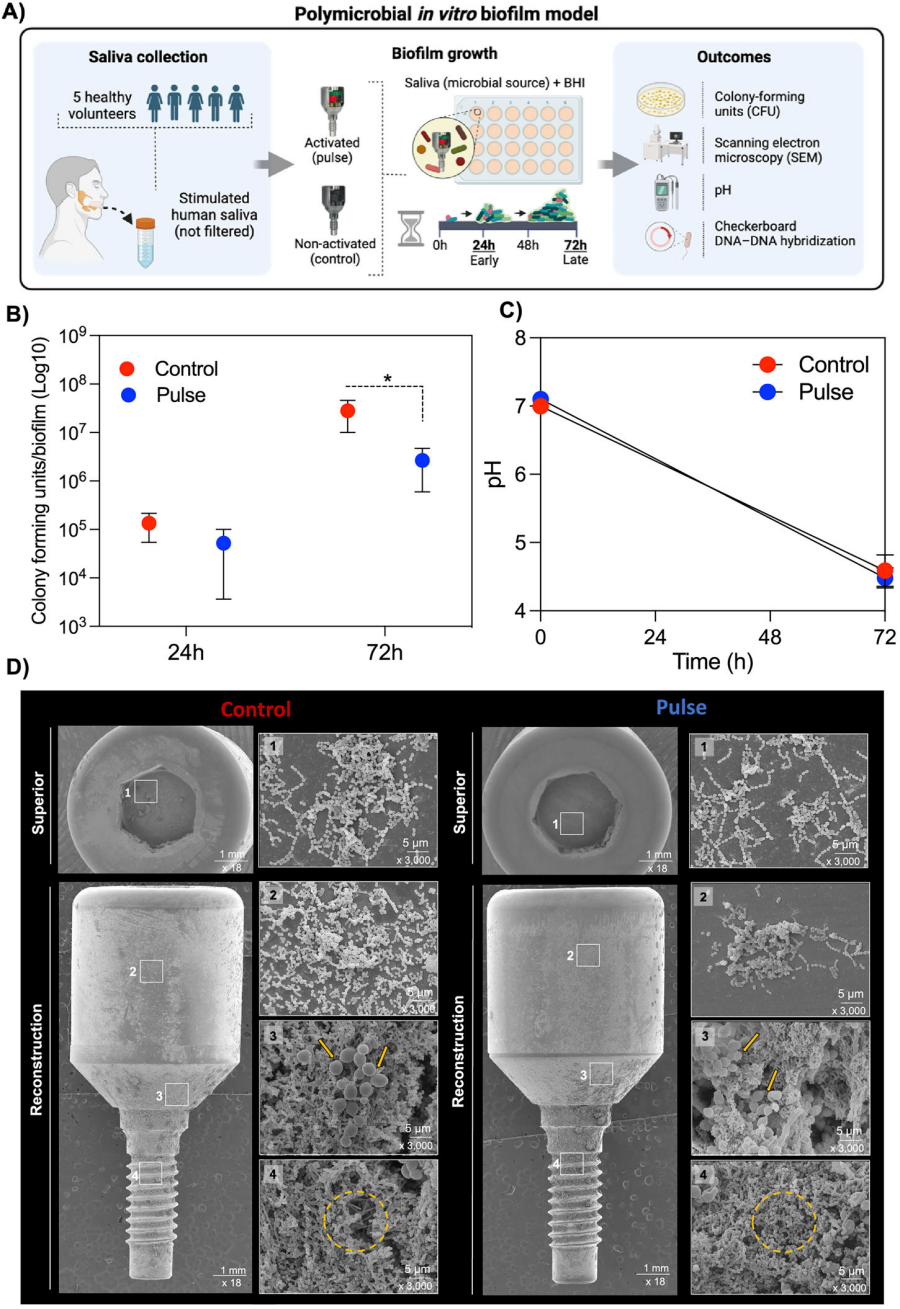
Pulsed electromagnetic field (PEMF) evaluated in terms of in vitro polymicrobial biofilm formation. **A** Stimulated human saliva (5 healthy volunteers) was used as microbial inoculum/source. Biofilms were incubated for 24 and 72 h and evaluated in terms of live bacterial cells count, structure, pH and composition. **B** Colony-forming units (CFU) for total live cell counts. Biofilm suspension was serially diluted and plated on Columbia Blood Agar (CBA) for measures. **C** Biofilm pH medium as bacterial metabolism analysis was evaluated at the beginning and after 72 h, using a pH meter. **D** Scanning electron microscopy micrographs of both groups after 72 h of biofilm formation. The circles and yellow lines represent microbial (streptococcal) aggregates or mixed-species aggregation on the surface. * Indicates statistical difference (*p* < 0.05) by t-test.

To evaluate the impact of PEMF on modulating the growth of polymicrobial biofilm-related infections and its microbial composition, a polymicrobial in vitro assay was conducted. Given that implant-related infections typically exhibit a polymicrobial profile and the oral environment harbors a diverse wide range of microbial species^6,10^, human saliva was used as the microbial inoculum/source for the biofilm assay (Fig. 3A). Furthermore, our analysis investigated both the initial/early (24 h) and mature/late (72 h) stages of biofilm formation to elucidate the effect on progressive microbial accumulation (Fig. 3A). PEMF demonstrated no effect on microbial accumulation in the early stages (24 h) of biofilm formation, displaying patterns similar to those of the control group (Fig. 3B). Nevertheless, during the late stages (72 h) of biofilm formation, a phase when the biofilm is expected to have the potential to trigger inflammatory processes, PEMF significantly reduced (p < 0.05) the microbial level cell counts, showing approximately 10 times (1-Log) lower counts than the control group (Fig. 3B). Notably, this reduction in microbial counts did not impact bacterial metabolism, as indicated by biofilm pH analysis (Fig. 3C). SEM micrographs revealed dense biofilm clusters in both groups after 72 h of biofilm accumulation (Fig. 3D), predominantly concentrated on the lower portion of the device where the topography exhibits greater irregularity. From the images, it is evident that biofilms were embedded in a dense extracellular matrix, with a high presence of coccoid-shaped microbial species. In the upper section of the device, the pulse group showed sparce bacterial clusters, in contrast with the control group, which displayed a higher microbial density (Fig. 3C). Therefore, the in vitro model revealed that PEMF may not prevent biofilm accumulation, but modulate its maturation, as no effect was found at 24 h, but a significant effect was observed after 72 h.

**Fig. 4:**
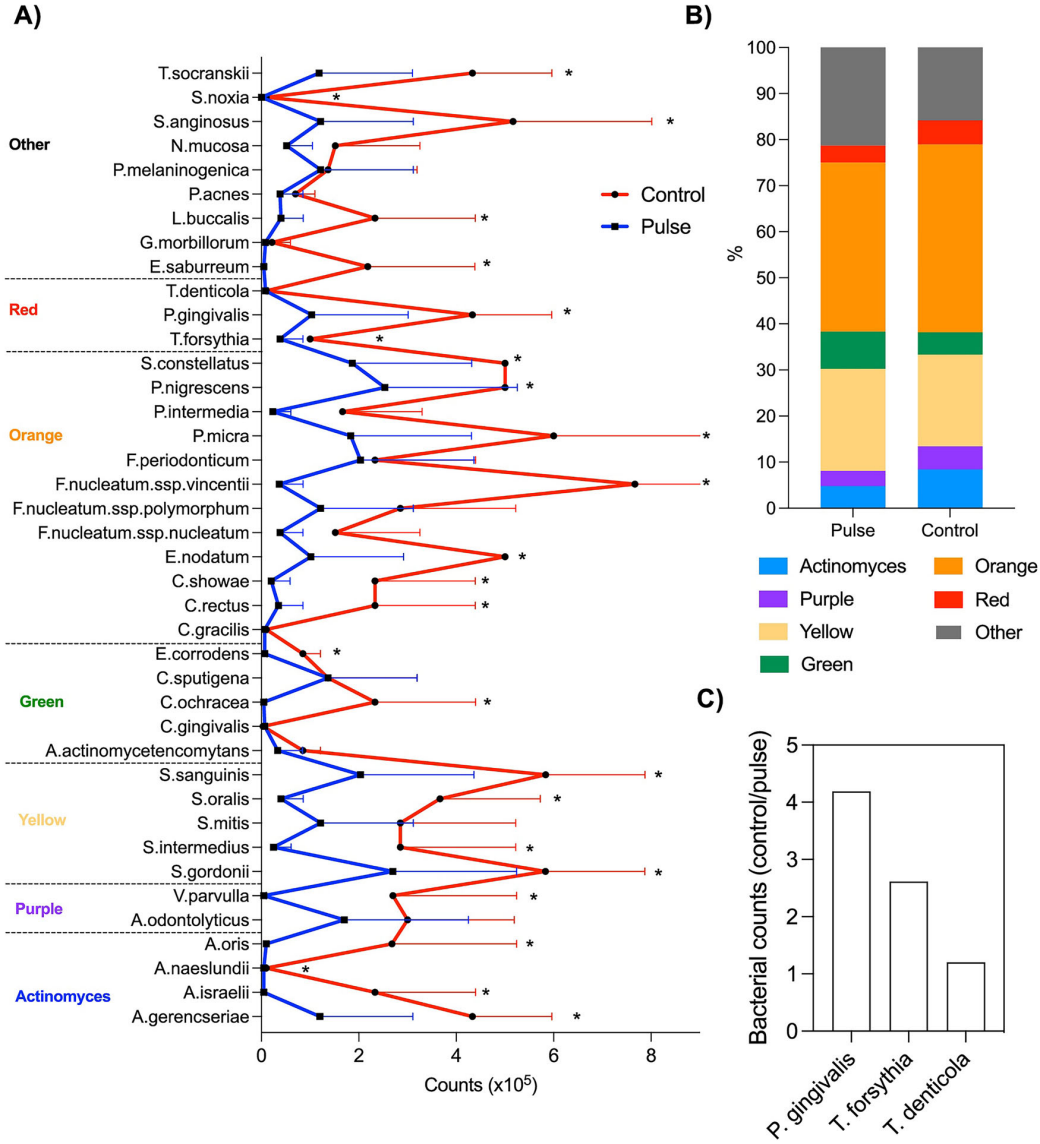
Pulsed electromagnetic field (PEMF) evaluated in terms of in vitro polymicrobial biofilm formation. Microbiological composition of in vitro biofilms was evaluated by checkerboard DNA–DNA hybridization technique, to assess the presence and levels of 40 bacterial species associated to dental implant-related infections. **A** Levels (x10^5^) of 40 bacterial species evaluated for both groups, pulse (activated PEMF) and control (non-activated) as average and standard deviation. **B** Periodontal microbial complexes by bacterial proportion. Bacterial species were grouped as previously described for microbial complexes related to oral infections. **C** Fold change of bacterial counts from control group divided by the counts in pulse group. Three bacterial species more associated with tissue damage in dental implant-related infections. During biofilm maturation and disease progression, initial colonizers start the process (complexes: *Actinomyces* – blue, yellow, green, and purple), followed by secondary colonizers (orange complex) that promote biofilm growth and create a suitable environment for the colonization of late colonizers (red complex), which are highly associated with tissue damage. * Indicates statistical difference (*p* < 0.05) by *t*-test.

The levels of 40 bacterial species closely associated with dental implant infections progression were examined. While no statistical differences (*p* > 0.05) were observed in total bacterial levels (CFU) between the groups (control vs. pulse) at 24 h, there was a noticeable trend towards reduced levels in the pulse group for certain species, such as *Fusobacterium periodonticum* (≈5 times lower in the pulse group) (Supplementary Fig. 1). At late stages of biofilm accumulation (72 h), PEMF exerted profound modulation on biofilm composition, significantly reducing (*p* < 0.05) the levels of 25 bacterial species (Fig. 4A), including putative anaerobic pathogens, such as *Porphyromonas gingivalis*, *Tannerella forsythia*, and *Treponema socranskii*. Considering the proportion of each bacterial species evaluated per total counts by sample, the periodontal microbial complexes encompassing the 40 bacterial species evaluated and divided according to their higher association with the pathogenesis of disease^34^, were not highly affected by PEMF, although higher proportion of orange and red complexes was found for the control group (Fig. 4B). However, among the three bacterial species from the red complex, the most pathogenic complex of oral infections, two of them showed increased levels for control group (Fig. 4C). *P. gingivalis*, one of the main pathogens associated with tissue damage in oral infections^35^, showed levels 4 times higher in the control group compared to the pulse group. Therefore, PEMF effectively controlled late polymicrobial biofilm accumulation in terms of microbial counts and modulated the microbial composition and levels to align with a health-associated profile.

### Pulse emitted by PEMF changes microbiome profile of biofilms accumulated in vivo in the oral environment

**Fig. 5:**
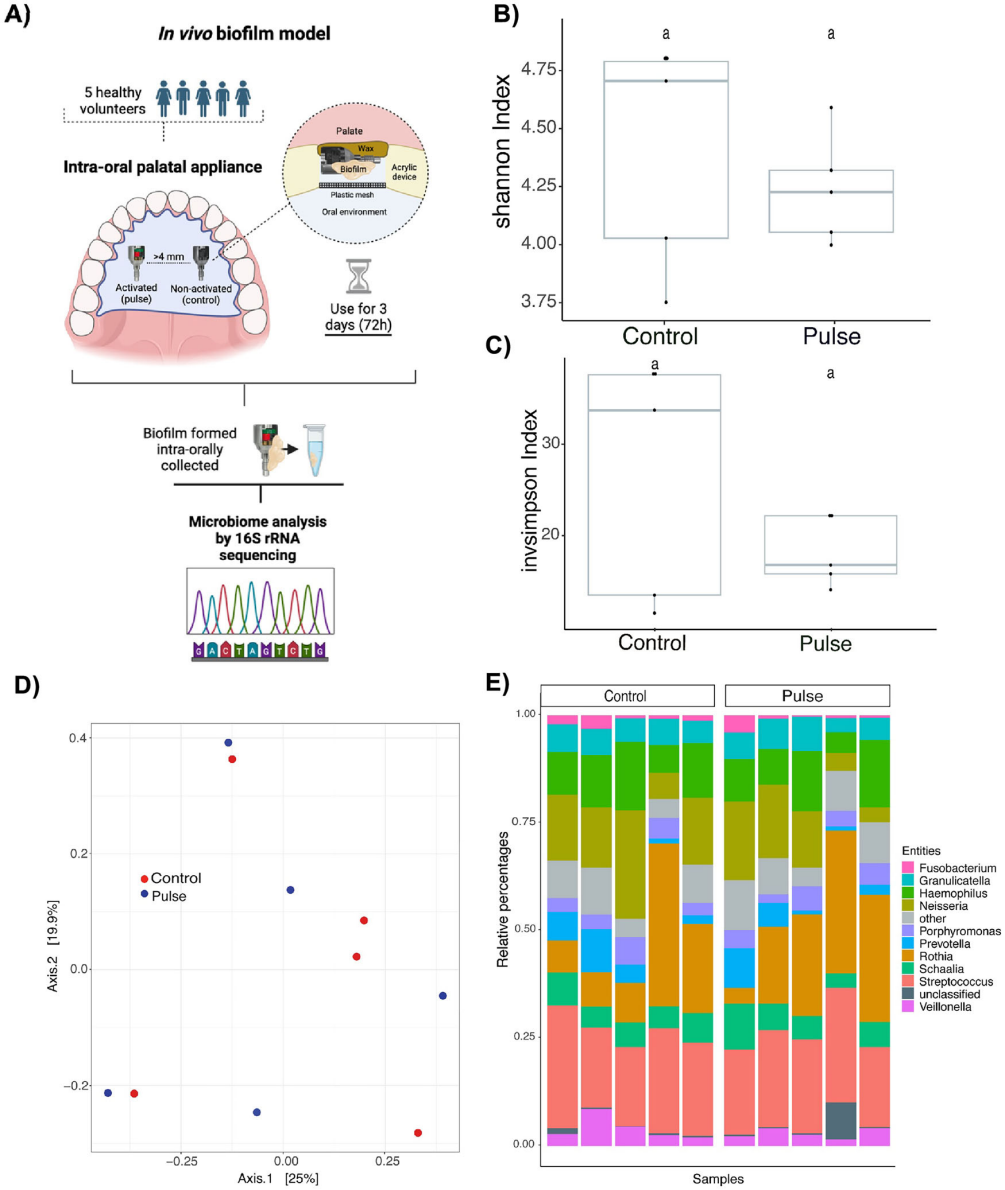
Pulsed electromagnetic field (PEMF) evaluated in terms of in vivo polymicrobial biofilm formation. **A** For this purpose, 5 healthy volunteers each wore one palatal appliance containing one activated PEMF (pulse) and one non-activated (control) device for 3 days to allow biofilm accumulation. Biofilm composition was evaluated by 16S rRNA sequencing for whole bacterial microbiome. **B** Alpha diversity analysis by Shannon Index and **C** Inv Simpson Index of sequenced samples. **D** Principal coordinates analysis (PCoA) using the Bray-Curtis distance function and ASV abundances. **E** Stacking bar charts showing dominant bacterial genus by sample and groups.

Our in vivo model explored the effect of PEMF on modulating microbial accumulation on the devices inserted in the oral cavity of patients, thereby simulating conditions close to clinical settings (Fig. 5A). For the first time, PEMF was evaluated in terms on microbial modulation in the oral environment. A total of 333 amplicon sequences variants (ASVs) were assigned taxonomy at the bacterial species level. Principal coordinate analysis (PCoA) (Fig. 5D) revealed highly heterogenous microbiome communities for both groups, with samples from both groups showing some proximity. In terms of richness, although alpha diversity analysis by the Shannon and Invsimpson Indexes showed no difference between the groups, there was a clear reduction in microbial diversity for the pulse group (Fig. 5B and C). In terms of the abundant microbial genus found, there was a predominance of *Streptococcus* and *Neisseria* genus for both groups (Fig. 5E). Although *Rothia* species were predominant for both groups, pulse group showed more samples with higher proportion of this genus (Fig. 5E).

**Fig. 6:**
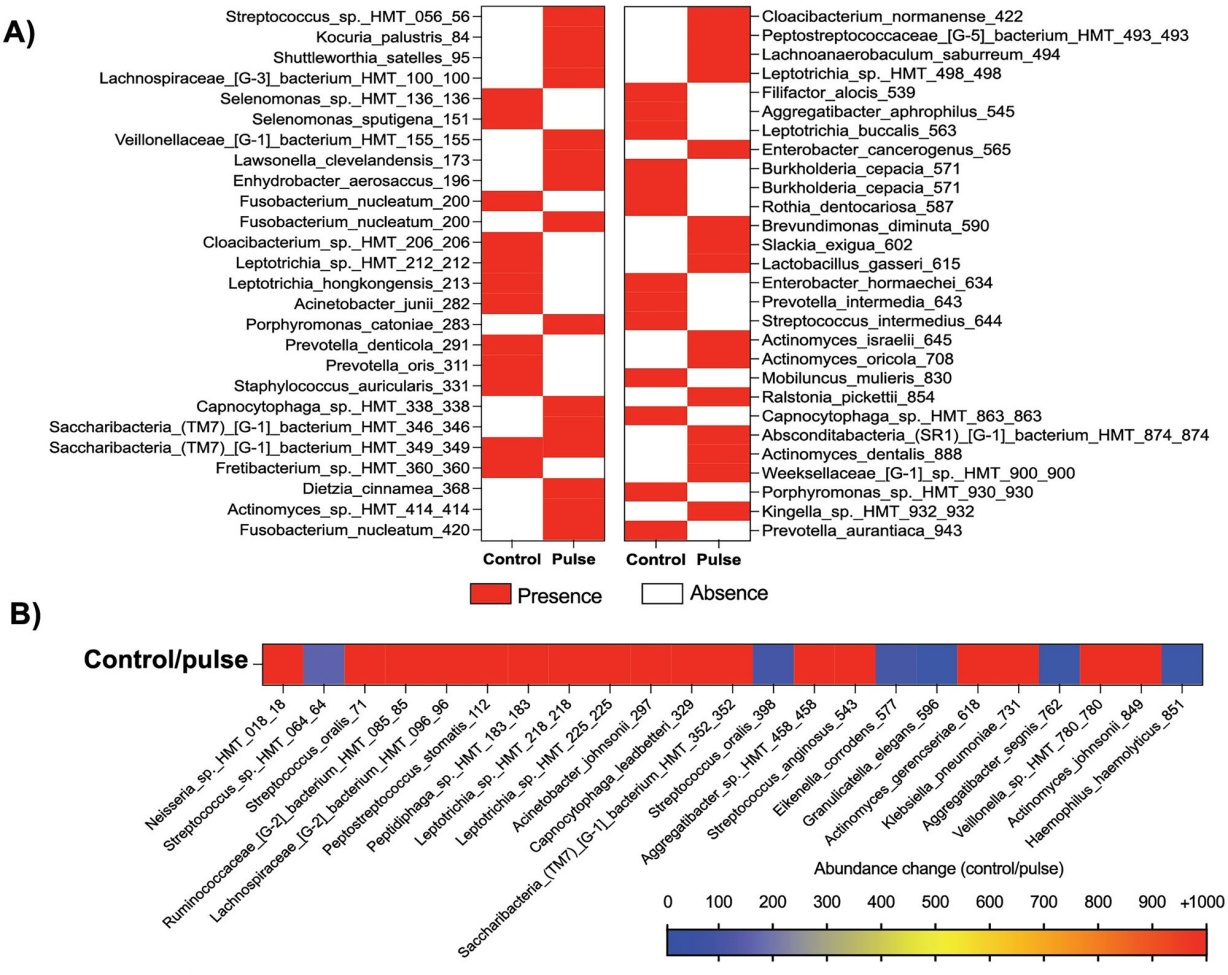
Pulsed electromagnetic field (PEMF) evaluated in terms of in vivo polymicrobial biofilm formation. For this purpose, 5 healthy volunteers each wore one palatal appliance containing one activated PEMF (pulse) and one non-activated (control) device for 3 days to allow biofilm accumulation. Biofilm composition was evaluated by 16S rRNA sequencing for whole bacterial microbiome. **A** Heatmap showing the absence or presence of bacterial species for control or pulse group. **B** Heatmap showing the bacterial species with increased level of abundance of at least 5 times in the control group, compared with the pulse group (ASV - control/pulse). Pulse – activated PEMF; Control – non-activated PEMF.

Given the increased richness for the control group and differences between the groups in terms of microbial ASVs, we explored the bacterial species that exhibited noteworthy increase in abundance for control group compared with the pulse group, as well as species present or absent in both groups. Twenty-five bacterial species were found only in the control group and thirty species only in the pulse group (Fig. 6A). Interestingly, four *Actinomyces* species (*A. dentalis, A. oricola, A. israelii, A. sp*.), which are highly associated with a healthy state, were found only in the pulse group, with no Actinomyces spp. found exclusively in the control group. Furthermore, five species of *Prevotella* and *Porphyromonas* spp. (*Porphyromonas sp., P. aurantiaca, P. intermedia, P. oris, P. denticola*), which are highly associated with oral disease and tissue damage, were found only in the control group (Fig. 6A). The abundance of 23 bacterial species increased by at least 5 times (5-fold change) in the control group compared with the pulse group, including putative pathogens such as *Aggregatibacter* (HMT-762, and 458) (Fig. 6B). Therefore, for late biofilms growing in the oral environment and considering the diversity of the entire oral microbiome, PEMF was able to modulate the microbial composition, mainly reducing the levels of important pathogens highly associated with dental implant infections or even the absence of some.

### Bacterial co-occurrence network analysis affected by PEMF

**Fig. 7:**
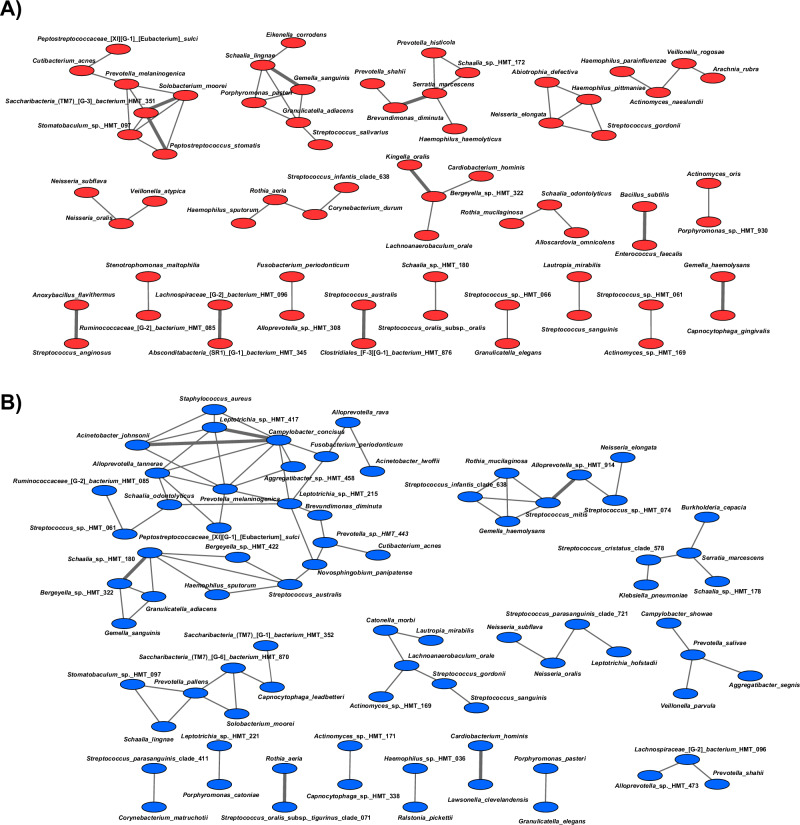
Co-occurrence networks evaluated the bacterial positive correlation. In all networks, each bacterium was represented by a node, and edges indicated the co-occurrence relationships between them. Bold lines indicated the interactions with significant co-occurrence. **A** Red - control group; (**B**) Blue—pulse group.

By constructing network structures, we investigated the co-occurrence relationships based on the 16S rRNA gene profiles of each group (control and pulse). Each bacterium was represented in these structures by a node, and edges represented the co-occurrence relationships. The clustering coefficients of the oral microbiome were 0.192 and 0.240 for the control and PEMF groups, respectively. We identified 65 and 76 nodes in the control and PEMF group, respectively. The numbers of statistically significant correlations were 10 and 6, and these correlations were group-specific (Fig. 7A and B). Importantly, although PEMF group showed more nodes, for control group important peri-implant pathogens showed more relationships, such as *Porphyromonas* spp (Fig. 7A).

### Metagenome prediction of the microbiome related to PEMF

**Fig. 8:**
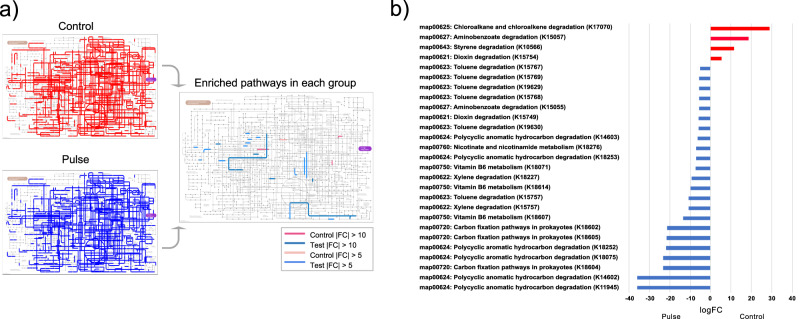
Evaluation of predicted pathways. **a** Predicted KEGG pathways were presented in any of the 5 samples for each group (left map) (Pulse - test / control), and only enriched pathways were in each group (right map). **b** Differential expression pattern of enriched pathways (|FC | > 5). FC – fold change.

PICRUSt2 analysis was performed to predict the relative abundance of gene functions in the oral and gut microbiomes. Although no specific pathways were found between control and PEMF groups, enriched pathways were detected in each group (Fig. 8A). Among the enriched pathways, chloroalkane and chloroalkene degradation, aminobenzoate degradation, styrene degradation, and dioxin degradation in control group were more enrich than PEMF group. On the other hand, polycyclic aromatic hydrocarbon degradation, carbon fixation pathways in prokaryotes, vitamin B6 metabolism, toluene degradation, xylene degradation, nicotinate and nicotinamide metabolism, and aminobenzoate degradation in PEMF group were more enrich than control group (Fig. 8B).

## Discussion

Implant-related infections represent prevalent conditions that often result in treatment failures, imposing significant costs on patients, clinicians, and healthcare systems^6^. Polymicrobial biofilms, which are central to triggering inflammatory cascades in peri-implant tissues, exhibit a diverse microbial composition and well-organized structure that promote antimicrobial resistance, making it a challenging task to eliminate this structure^36^. Coupled with the intricate structure of implant devices, the absence of effective therapies for dental implant-related infections is evident^15^. Currently, no FDA-approved treatment for implant-related infections has been reported. In this context, PEMF technology has explored bioelectromagnetic phenomena to modulate biological responses, including microbial accumulation^29^. Here, for the first time, we investigated the role of PEMF in controlling biofilm accumulation and modulating its microbial composition using polymicrobial models that consider the human microbiome, specifically the oral environment, which is the second largest microbiome in the human body. Our findings indicate that PEMF activation did not affect the material surface properties, which are designed to withstand the environmental challenges in the oral cavity, and facilitated salivary protein adsorption, a key host-response mechanism that modulates subsequent biological processes^37,38^. In addition to demonstrating the potential of PEMF technology for biomaterials manufacturing, the main strength of our study lies in its microbiological outcomes. PEMF effectively reduced late microbial accumulation and considerably modulate the microbial composition of biofilm by reducing key putative pathogens strongly associated with implant infections and tissue damage. These findings were further validated by our in vivo model, where biofilm accumulation occurred in the oral cavity of patients, mirroring clinical conditions. Moreover, PEMF altered bacterial interactions and promoted specific bacterial pathways. Therefore, we have unveiled a potent antimicrobial strategy for controlling and treating implant-related biofilms, paving the way for promising avenue to tackle these infections.

The same PEMF device used in this study has been undergone clinical testing, which has demonstrated its effectiveness in enhancing dental implant stability, encouraging implant-bone contact, and managing bone loss in patients with implant-related infections^26,39,40^. Indeed, clinical observations have shown that PEMF technology fosters bone regeneration in different conditions, including cervical fusion^41^, mandibular fracture^42,43^, and bone formation in women with postmenopausal osteoporosis^44^. The use of PEMF has consistently demonstrated increased bone formation and density, along with a faster healing process^42^. Moreover, we found increased protein adsorption for the PEMF group, signifying an important and positive biological response in the interaction between implant devices and human body^37^. Dental implant-related infections, particularly peri-implantitis, are characterized by progressive bone loss. Therefore, PEMF emerges as a powerful strategy to control and modulate the etiological factor - biofilm accumulation - while concurrently promoting tissue regeneration to restore health states. Further clinical trials should delve into and comprehensively investigate both effects on disease conditions, examining the effectiveness and durability of outcomes over extended periods, and elucidating its mechanisms. Moreover, the direct and modulatory effects of this technology on immune responses would provide valuable insights and should also be investigated.

Our results suggest that PEMF may not prevent polymicrobial adhesion. Instead, this technology appears to exert a modulatory influence on biofilm progression and microbial interactions. Notably, we observed reduced bacterial cell counts only in 72 hour biofilms, with no significant difference found the 24 hour time point. The lack of effect at early stages of biofilm formation may also explain the absence of an impact on protein adsorption, which was not affected by PEMF. While PEMF has shown some ability to inhibit biofilm accumulation and reduce 24 hour biofilm biomass by half in monospecies biofilms, such as *S. epidermidis*^29^, polymicrobial biofilms may present distinct features that promote microbial growth, tolerance, and persistence. These features include reprogramming of transcriptomic and metabolic apparatuses, as well as increased extracellular polymer synthesis^45^. In fact, previous studies evaluating the use of PEMF technology to control biofilm growth and test its antibacterial properties have been conducted using in vitro models with specific microbial species^29–31^. Overall, these studies predominantly used single microbial species, tested PEMF technology on preformed biofilms, assessed live cells in biofilms through absorbance measurements or even combined the PEMF with nanoparticles treatment (Khan et al.^29,30^), explains the antimicrobial ability to eliminate more than half of the biofilms.

Interactions between different microbial species, as well as their metabolites, play a crucial role in microbial physiology, community structure, and susceptibility to antimicrobial strategies^46^. The observed reduction in bacterial levels at 72 h may be attributed to the PEMF effect on modulating microbial interactions, potentially suppressing some bacterial species growth and delaying co-aggregation processes. After initial colonizers adhere to the implant surface and create a suitable environment for other species, the co-aggregation process starts, promoting the interaction and colonization of putative pathogens (late colonizers)^7^. Since our in vitro model showed no difference in terms of live cell counts and microbiological compositions at early stages (24 h) but a strong effect, mainly on composition, at later stages (72 h), it is clear that PEMF modulated the biofilm dynamics during maturation, particularly microbial interactions and growth. Considering our biofilm model, which mimicked the whole microbial diversity in the oral environment (using human saliva as the microbial source) and the evaluation at different stages of biofilm growth, these findings highlight for the first time the ability of PEMF technology to modulate biofilm maturation during its growth, reducing the levels of important pathogens associated with dental implant infections. Previous evidence tested the PEMF device on an in vitro oral biofilm model, but using specific bacterial species (31 species standardized at specific loads)^33^. However, this does not adequately represent the oral microbiome or in vivo biofilm formation, particularly considering that at early stages, putative pathogens are present at lower levels, but biofilm maturation provides a suitable environment for the overgrowth of these species. In our study, we mimicked the oral microbiome using human saliva as the inoculum and allowed normal biofilm growth and interactions. Importantly, further studies should investigate PEMF therapy at distal sites on the implant body, including subgingival surfaces, to assess reduction in microbial load and pathogen abundance.

The modulatory effect of PEMF on the microbiological composition of biofilms was found in both in vitro and in vivo models. Late-stage biofilms (72 h), representing the critical stage triggering inflammatory processes in implant-related infections^8^, were particularly affected. Notably, the abundance of *Porphyromonas* species was significantly reduced by PEMF in both biofilm models, with some species even absence in the PEMF group in the in vivo model. *P. gingivalis*, in particular, is recognized as one of the key pathogens highly associated with peri-implantitis disease and tissue damage^47^. Moreover, the putative pathogen *T. forsythia*, known for its association with peri-implant tissue destruction^48^, also exhibited reduced levels in response to PEMF. The effect of PEMF led to the reduction of 23 or more bacterial species in our models, demonstrating a potent modulatory effect. Moreover, the in vivo model showed that some important disease-associated species were found only for the control group, such as some *Prevotella* and *Porphyromonas* spp., and health-associated species, such as some *Actinomyces* spp., were found only in the pulse group, suggesting a trend towards a microbial profile associated with health for PEMF. Importantly, previous evidence has evaluated the antimicrobial effect of PEMF using only in vitro models, without testing even in animal models^29,30^. Here, we took the next step by testing this technology in humans, using an in vivo model where the devices were inserted into the oral cavities of patients and kept for three days. This was possible because the device had been previously tested in clinical trials focusing on its effect on bone formation and regeneration^26,39,40^. Therefore, we reproduced a clinical setting in a controlled condition, demonstrating a promising effect that can now be confirmed by randomized clinical trials before being recommended by the industry for this purpose. Importantly, the use of a palatal appliance for 3 days was based on previous evidence using the same model, which demonstrated that microbial levels and biofilm composition on titanium surfaces were similar to those found clinically in patients with peri-implant disease^49^.

**Fig. 9:**
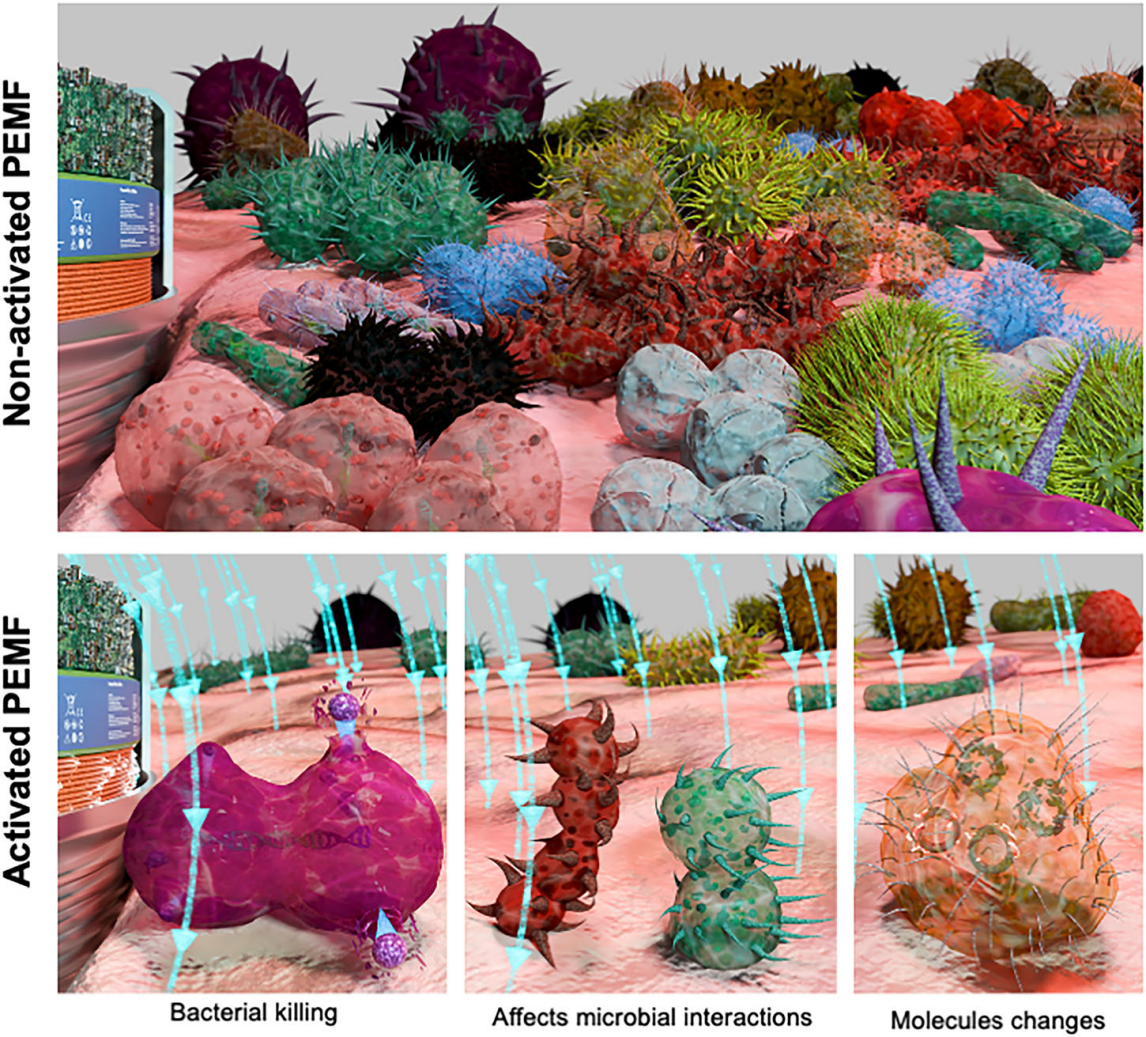
Hypothesis of mechanisms by which Pulsed Electromagnetic miniaturized device (PEMF) modulates biofilm accumulation. Non-activated PEMF allows polymicrobial biofilm accumulation with increased pathogens growth. In contrast, activated PEMF showed reduced polymicrobial biofilm formation. Some mechanisms has been raised based on our findings and previous evidence: (1) electroporation effect on microbial cell membranes resulting in bacterial killing; (2) modulation of bacterial-bacterial interactions due electrostatic forces changes; (3) changes in cell-wall surface molecules and surface charges modulating microbial growth.

Although the mechanism by which PEMF modulates microbial interaction and reduces biofilm accumulation is not fully understood, some hypotheses have been raised (Fig. 9). Electrostatic forces are responsible for mediating bacterial-bacterial interactions and surface attachment, and their modulation may be influenced by PEMF activation^50^, particularly affecting Gram-negative species owing to the distinct nature of their membranes and charges^51^. PEMF emission has been associated with changes in cell-wall surface molecules and surface charges^27^, which are critical parameters governing microbial interactions and growth. While the field created by PEMF may impact all cellular components to some extent, it is anticipated to have a higher effect on extracellular molecules since the PEMF energy is attenuated after penetrating the membrane^52,53^. In this context, it is crucial to further investigate the potential impact of PEMF on the extracellular matrix content, as it plays a significant role in protecting microbial cells and facilitating microbial growth. Moreover, PEMF activation may induce an electroporation effect on microbial cell membranes^29^ potentially resulting in bacteriostatic or bactericidal effects. Importantly, although some studies have reported magnetic field generating heat to promote bacterial killing on metallic devices^54^, the PEMF device tested here was not expected to generate no heat effects. This is because it operated at a low frequency was (10–50 kHz), which is unlikely to compromise the surrounding tissues. Further research is warranted to elucidate these mechanisms and their implications.

Here, we also confirmed some of the hypotheses raised regarding the modulatory effect of PEMF on oral biofilms. The network analysis emphasized the effect of PEMF on bacterial interactions, based on the co-occurrence and abundances of bacterial species for all samples. In the control group pathogenic species such as *Porphyromonas spp* showed more connections (3 nodes) with other species compared to the pulse group (1 node). The biofilm accumulation is driven by co-aggregation processes between initial and late colonizers^8^. The absence and presence of some species in both groups, and the higher abundance of 23 bacterial species in the control group, indicate differences in microbial composition and levels, which may have affected these co-aggregation processes and biofilm pathogenicity. Since bacterial-bacterial interaction is also mediated by electrostatic forces, which are directly affected by PEMF activation^50^, these changes on cell wall surfaces may have impaired some microbial cells’ interactions (as shown by fewer nodes for PEMF), leading to reduced levels or even the absence of some bacterial species (as shown by our microbiome data). Additionally, the clustering coefficients of the PEMF group, a tool for understanding the structure of complex networks, were greater than those of the control group, suggesting that the networks in the PEMF group were more complex. To protect against the invasion of pathogens, the gut microbiome constitutes a complex network of microorganisms^55^. These results mean that the networks of the PEMF group were more robust than those of the control group. Interestingly, lower diversity and complexity have been found in disease-associated biofilms in dental implant-related infections^56^.

The second mechanism found of how PEMF modulated the biofilm composition is related to bacterial pathways, according to bacterial genome and its abundance. PEMF generated a stressful environment as reflected on microbial pathways, with toluene degradation emerging as a pathway increased for biofilms exposed to PEMF. Since the toluene biodegradation is a carbon and energy source for microbial cells^57^, the increased pathway may represent a survival mechanism triggered under PEMF conditions. Another important pathway increased for PEMF was polycyclic aromatic hydrocarbon (PAH) degradation, mainly considering that these compounds pose a high risk to health^58^. Importantly, *Actinomyces* spp., a health-associated oral bacterium, has been recognized as a good metabolizer of PAH^58^, and these species were highly found mainly in the PEMF group. Therefore, we showed for the first time two mechanisms of how PEMF modulates polymicrobial in vivo biofilms: changing bacterial interactions and modulating the microenvironment, which affects bacterial behavior and pathways.

Although PEMF exhibited a notable modulatory influence on biofilm composition, further assessment in clinical studies is necessary to understand its effect on biofilm virulence and its potential impact on disease control, particularly in peri-implant disease settings or in permanent abutments that have an external structure capable of resisting oral environmental challenges, including occlusal loads. While PEMF application may reduce the reliance on systemic antibiotics for treating implant-related infections, a more comprehensive comparison, including the combination of the device with mechanical debridement or chemical strategies, is warranted. Given the role of *Candida* species in implant-related infections^59^, exploring the effect of PEMF on fungal diversity could unveil its application for diverse pathological conditions. Moreover, different time points also need to be evaluated to more accurately reflect the true effect of PEMF technology in modulating microbial interactions and profiles during biofilm maturation. Despite inherent limitations, we showed for the first time the potent capacity of PEMF to control and modulate polymicrobial biofilms associated with dental implant infections.

PEMF technology successfully suppressed the growth of pivotal putative pathogens related to dental implant infections, while modulating biofilm composition toward a health-associated profile. Importabtly, this was achieved without compromising the surface properties and electrochemical behavior of the device. Therefore, PEMF emerges as a promising and effective strategy for controlling implant-related infections. Urgent evaluation through clinical trials is warranted to further validate PEMF as an emergent approach to managing this prevalent and challenging condition.

## Methods

### Miniaturized PEMF biomedical device

Specimens used were miniaturized PEMF devices (Magdent®, Tel Aviv, Israel) shaped like a healing abutment (healing cap) for dental implants with a 1.25 mm (0.05”) hexagonal socket. The PEMF device consisted of a battery (source of power), an electronic component and a coil encased within a Ti6Al4V (Grade 5) structure. The electromagnetic field was activated within a 2 mm radius, exposure ratio of 1/500–1/5000, intensity ranging from 0.05 to 0.5 mT, and a frequency between 10 and 50 kHz. The average frequency of devices used was estimated at 35.3 Hz using an oscilloscope. Once the device is activated, it generates the PEMF throughout the coil. For activation, the device was inserted into an activator apparatus that uses a magnetic mechanism to start the battery. After battery activation, PEMF generated the electromagnetic field for 30 consecutive days. Therefore, the exposure time is determined by the battery life, which allows the device to operate for up to 30 days. In our study, in the “pulse group” where the device was activated, the exposure time matched the biofilm formation period (up to 72 h) or the duration of the assays, which was less than 30 days. During the manufacturing process, the device integrates a coil, ferrite core, battery, and a PCB with microchips and embedded software into the hollow healing abutment. These components regulate the pulsed electromagnetic field, including its waveform and frequency. To ensure precision and functionality, the device is tested using an electric tester connected to an oscilloscope, which displays the sinusoidal waveform along with its power output, confirming whether the device operates within the predefined frequency range.

The experimental group consisted of samples with activated electromagnetic potential (pulse), while the control group consisted of similar samples that remained inactive (control). Substrates were sterilized by γ radiation. Activated PEMF was used as experimental group (pulse). Non-activated PEMF was used as control. For surface properties evaluation (roughness, wettability and chemical composition), PEMF device after activation phase (after 30 days of activation) was used as control to evaluate the effect of pulse emission on surface’s properties.

### Surfaces roughness and wettability

Surface roughness parameters (average roughness, Ra; root mean-square-average, Rq; average maximum height of the profile, Rz; and maximum height, Rt) were measured by topographic profiles acquired using a profilemeter (cut-off of 0.25 mm at 0.05 mm/second over 12 sec) on the upper portion of the device (Dektak D150; Veeco system, Plainview, USA)^49^. For water wettability assessment, the contact angle was estimated. Thus, the sessile drop (deionized water droplets – 10 μL) technique using a goniometer (Rame–Hart 100–00, New Jersey, USA) and a software supplied by the system manufacturer were used. The Young equation was used to measure the contact angle between water and the material. Surface roughness and wettability were evaluated before and after 30 days of pulse activation.

### Surface chemical composition

The Surface chemical composition was evaluated by energy dispersive spectroscopy (EDS). Composition was evaluated before and after 30 days of pulse activation. For this purposes, a detector attached to the scanning electron microscopy apparatus (SEM; JEOL JSM-6010LA, JEOL, Tokyo, Japan) was used (beam energy of 5.0 and 10.0 keV)^49^.

### Electrochemical assessment

To assess whether pulsed electromagnetic fields had any influence on the corrosion performance of healing abutments, electrochemical tests were conducted to analyze the corrosion stability of pulse and control healing in artificial saliva^60^. All measurements were taken by a standardized method of three-electrode cells, as required by the ASTM International specifications (G61–86 and G31–72). A saturated calomel electrode (SCE) was used as a reference electrode, a graphite rod as the counter electrode, and the exposed area of healing as the working electrode. First, to standardize the oxide layer of the healing, a cathodic potential (-0.9 V vs SCE) was applied for 10 min. Thereafter, the open circuit potential (OCP) was performed for 3600 s to evaluate the free corrosion potential of the material. Then, electrochemical impedance spectroscopy (EIS) was performed at frequencies of 100 kHz to 5 mHz with amplitude of the sinusoidal voltage signal of 10 mV and OCP as the initial potential^61^. The Echem Analyst software (Gamry Instruments, Warminster, USA) was used to analyze EIS data and to construct Nyquist, Bode (|Ζ|), and phase angle plots based on the imaginary (Ζ”) and real (Ζ’) components of impedance. Therefore, an equivalent electrical circuit was fitted to quantify the corrosion process in the passive/oxide film formation. Finally, the material was polarized from -0.8 to 1.8 V at a scan rate of 2 mV/s to draw the potentiodynamic polarization curves^62^. The Tafel extrapolation method (Echem Analyst Software, Gamry Instruments, Warminster, USA) was used to achieve the E_corr_ (corrosion potential), *i*_corr_ (corrosion current density), Tafel cathodic (βc) and anodic (βa) slopes, and *i*_pass_ (passivation current density). All electrochemical tests were repeated at least five times (*n* = 5) to ensure reliability and reproducibility.

### Biological response—protein adsorption

To evaluate the effect of PEMF activated on total protein adsorption, the substrate was exposed to human saliva protein adsorption, and the results were compared with those of the non-activated group. For the salivary pellicle adsorption assay, a pool of stimulated human saliva (5 healthy volunteers) was used (Souza et al.^37^). For this purpose, stimulated saliva was collected at least 2 h after the volunteers had eaten and brushed their teeth following the previous protocol^37,63^. Then, the saliva pool was centrifuged (10 min, 3,800 *g*) and filtered (0.22 μm) and substrates were immersed in 2 mL of saliva in a 24-well plate, at 35°C. Following salivary pellicle maturation, substrates were washed three times with 0.9% saline solution to remove non adsorbed proteins. Subsequently, they were vortexed, and sonicated to remove all adsorbed proteins. Total protein was quantified by using the bicinchoninic acid method (BCA Kit; Sigma-Aldrich, St. Louis, USA)^64^.

### Microbiological assay

#### In vitro biofilm

To test the effect of activated PEMF on progressive microbial accumulation, an in vitro polymicrobial biofilm model was used. For this purpose, stimulated human saliva (5 healthy volunteers) was used as microbial inoculum/source for biofilm growth to reproduce the entire oral microbiome, as standardized by previous studies^65^^,^^66^. Saliva from healthy volunteers presents similar microbial outcomes compared to biofilms from patients with peri-implant diseases as microbial inoculum, as shown in a previous publication^65^. Thus, substrates (pulse and control – non-activated) (*n* = 6 samples per group) were incubated in 12-well plates with 2 mL of a solution containing salivary microbial inoculum + BHI medium (Becton-Dickinson, Sparks, USA) (10:1 v/v) for early (24 h) and late (72 h) biofilm formation at 37°C under 10% CO_2_. The medium was supplemented with sucrose (10:1 v/v) to promote the growth of oral putative pathogens due to its effect on biofilm structure and extracellular matrix, as previously described^65,67^. After biofilm formation, substrates were washed (3x – 0.9% saline solution), vortexed (30 s), and collected for analysis.

For total live cell counts, vortexed and sonicated biofilm suspension was serially diluted and plated on Columbia Blood Agar (CBA) for measurement of colony-forming units (CFU). Undisrupted biofilms on substrates were used to visualize biofilm morphology and distribution on the PEMF device. For this purpose, scanning electron microscopy (SEM; JEOL JSM-6010LA, JEOL, Tokyo, Japan) was used. Biofilm pH medium was evaluated (pH meter) at the beginning and after 72 h as indirect evaluation of bacterial metabolism.

The microbiological composition of in vitro biofilms was evaluated by the checkerboard DNA–DNA hybridization technique, to assess the presence and levels of 40 bacterial species associated with the progression of dental implant-related infections^34,68^. The 40 bacterial species evaluated was also showed as their periodontal microbial complexes. The bacterial levels identified were compared to standard controls^68^. These microbial complexes, also known as clusters, have been used for more than two decades to categorize groups of bacterial species associated with the transition from health to disease in the oral cavity, primarily in biofilm-related diseases affecting the tissues surrounding tooth and implant surfaces. The bacterial species are grouped according to their co-occurrence and the roles they play in health and disease and are shown by different colors. During biofilm maturation and disease progression, initial colonizers start the process (complexes: *Actinomyces* – blue, yellow, green, and purple), followed by secondary colonizers (orange complex) that promote biofilm growth and create a suitable environment for the colonization of late colonizers (red complex), which are highly associated with tissue damage. These complexes have been widely used by both in vitro and in vivo models to describe dental implant-related infections^65,67,69^.

#### In vivo model

To accurately assess the impact of PEMF on modulating oral microbial adhesion and accumulation on the devices, while accounting for all factors influencing microbial attachment in the oral environment, we employed an in vivo model (approved by the local Research and Ethics Committee - protocol 54251721.6.0000.5506, written consent was obtained from all patients). To this end, the same 5 healthy volunteers (3 men and 2 women) each wore a palatal appliance for 3 days. Therefore, we conducted an in vivo model with humans. Sample size considered similar number of volunteers of previous study testing antimicrobial protocols on implant devices^17,66^. Importantly, volunteers were selected based on previous established inclusion criteria: over 18 years old (18–35 years old), good systemic and oral health, normal stimulated salivary flow rate (> 0.7 mL/min), no recent antibiotic intake within the month prior to the study, being nonsmokers, and not using mouthwash^67^. The selected volunteers did not have dental implants. The appliance included one activated PEMF (pulse) and one non-activated (control) device (*n* = 5 samples per group). The distance between substrates within same appliance was set at twice the pulse radius (2 mm). The devices were exposed extra-orally, 4 times/per day, to a 20% sucrose solution to promote the growth of pathogens highly associated to dental implant-related infections, thereby mimicking a biofilm composition with bacterial loads similar to those found in diseased patients as reported in clinical trials (Shibli et al.; Souza et al.^67^). Following the experimental phase, substrates were immersed in tubes containing TE solution, vortexed and the biofilm suspensions were evaluated using 16S rRNA sequencing to analyze the entire bacterial microbiome composition.

For this purpose, genomic DNA were extracted using the ZymoBIOMICS®-96 MagBead DNA Kit (Zymo Research, Irvine, USA). The quantity and quality of DNA were evaluated using absorbance (A260/A280) by NanoDrop™One/One C (Thermo Fisher, Waltham, USA); amplification by RT-PCR OPUS (Bio-Rad, Berkeley, USA); and qualification of fragments during library preparation by 4200 TapeStation (Agilent, Santa Clara, USA). Bacterial 16S ribosomal RNA gene sequencing was performed using the Quick-16S™ NGS Library Prep Kit (Zymo Research, Irvine, USA). The V3-V4 region of the 16S rRNA gene was amplified. The final libraries were sequenced on Illumina® MiSeq™ with a v3 reagent kit (600 cycles). 16S rRNA amplicon sequence variants (ASV) were inferred from raw reads using DADA2^70^ and counts normalized using Metacoder^71^. Taxonomic assignment was conducted using a custom DECIPHER classifier (Murali et al.) generated from the 16S rRNA gene database of the Human Oral Microbiome Database (eHOMD)^72,73^. Alpha diversity metrics and beta diversity plots were generated using Metacoder.

#### Network and pathways analysis

To evaluate the bacterial correlations and functional composition, Qiime2 (version 2022.2) with DADA2 was used for ASVs measures^74^. The taxonomic classification of ASVs was performed using the qiime feature-classifier classify-sklearn based on the eHOMD database. Co-occurrence coefficients were calculated based on the using taxonomy data the SparCC program^75^. Bacterium pairs with SparCC values ≥ 0.95 were considered to have a co-occurrence relationship with a positive correlation. The networks were visualized using version 2.8 of the Cytoscape software^76^. The median correlation of each pairwise comparison and each correlation were estimated by 10 and 500 iterations^77^. The analysis of potential functional genes was performed using the q2-picrust plug-in (version 2021.11) based on the taxonomy data obtained from QIIME2 and Kyoto Encyclopedia of Genes and Genomes (KEGG) database^78^. The predicted read abundance was normalized by reads per million mapped reads. The pathways were visualized using iPath3.0^79^.

### Statistics

SPSS 20.0 software (IBM Corp., Armonk, USA) and Prism 10.0 (GraphPad, Boston, USA) were used for statistical analyses and a significance level of 5% was adopted. T-test was applied to analyze the data. In network analysis, the statistical significance threshold was *p* < 0.05 and *q* < 0.1 using PseudoPvals in SparCC and Benjamini-Hochberg’s procedure, respectively^21,80^.

## Supplementary information

Supplementary information

## Data Availability

The datasets (microbiome data) generated and analyzed during the current study will be available in a public repository when accepted (10.7910/DVN/MB84XU).
