# Supplementary material for: Microbiome modulation of implant-related infection by a novel miniaturized pulsed electromagnetic field device
Source: NPJ Biofilms Microbiomes. 2025 Feb 26;11:36. doi: 10.1038/s41522-025-00667-0 (PMC11865433; doi:10.1038/s41522-025-00667-0)

Microbiome modulation of implant-related infection by a novel miniaturized pulsed electromagnetic field device

Table 1 - Means and standard deviations of electrical parameters (polarization resistance – R_{ptot} and capacitance - Q_{tot}) obtained from the equivalent circuit models of control and pulse groups.

Groups	R_{pout} ($M\Omega \cdot cm^2$)	R_{pin} ($M\Omega \cdot cm^2$)	R_{tot} ($M\Omega \cdot cm^2$)	Q_{out} (S*s^a) × 10^{-6}	Q_{in} (S*s^a) × 10^{-6}	Q_{tot} (S*s^a) × 10^{-6}	η_1	η_2	W_{diff} (S*s^(1/2)) X 10^{-6}	$X^2 \times 10^{-3}$
Control	36.43 (±44.14)	19.91 (±19.49)	56.34 (±63.63)[a]	1267.09 (±2044.17)	8.36 (±5.92)	1275.45 (±2050.09)[a]	0.77 (±0.09)	0.86 (±0.16)	5.24 (±0.10)	8.45 (±7.60)
Pulse	1.64 (±2.21)	14.69 (±11.36)	16.34 (±13.57)[a]	7374.28 (±137.1)	7.04 (±7.95)	7381.32 (±142.71)[a]	0.83 (±0.09)	0.63 (±0.21)	1.38 (±1.26)	3.21 (±4.33)

Different letters indicate statistically significant differences among the groups ($p < 0.05$). R_p = polarization resistance, Q = capacitance. n and $X^2{}_x$ 10^{-3} obtained from EIS (goodness-of-fit on the order of 10^{-3}).

Table 2 - Mean and standard deviation (in parenthesis) values of electrochemical parameters (E_{corr}, i_{corr}, βa, -βc, i_{pass}, and corrosion rate) obtained from the control and pulse groups.

Groups	E_{corr} (mV) vs. SCE	i_{corr} ($nA \cdot cm^{-2}$)	βa (V/dec^{-1})	-βc (V/dec^{-1})	I_{pass} ($nA \cdot cm^{-2}$)
Control	-238.62 (±162.70)[a]	12.88 (±10.71)[a]	1.07 (±1.08)	0.56 (±0.18)	84.26 (±45.68)[a]
Pulse	-288.16 (±162.28)[a]	28.34 (±25.96)[a]	2.64 (±2.68)	0.72 (±0.36)	50.22 (±20.96)[a]

Different letters indicate statistically significant differences among the groups ($p < 0.05$)

Figure 1 - Pulsed electromagnetic field (PEMF) evaluated in terms of in vitro polymicrobial biofilm formation (24h). Microbiological composition of in vitro biofilms was evaluated by checkerboard DNA–DNA hybridization technique, to assess the presence and levels of 40 bacterial species associated to dental implant-related infections. (A) Levels (x10^5) of 40 bacterial species evaluated for both groups, pulse (activated PEMF) and control (non-activated).

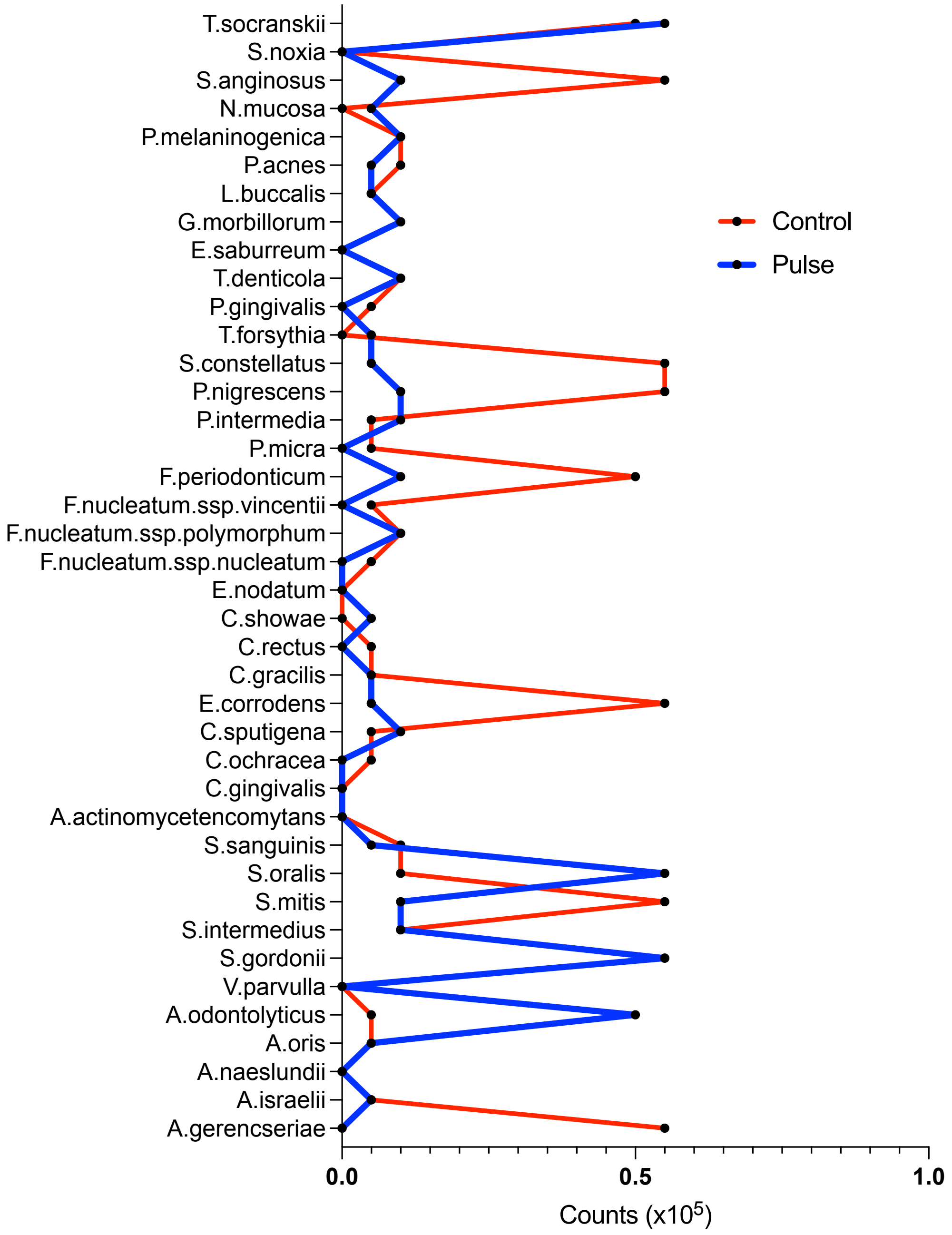

Supplement: Supplementary file 1 — Supplementary information [file 41522_2025_667_MOESM1_ESM.pdf]
